# Effect of intensive training in improving older women's knowledge and support for infant vaccination in Nigerian urban slums: a before-and-after intervention study

**DOI:** 10.1186/s12889-021-10310-0

**Published:** 2021-02-02

**Authors:** Folusho Mubowale Balogun, Olayinka Samson Bamidele, Eniola Adetola Bamgboye

**Affiliations:** 1 Institute of Child Health, College of Medicine, University of Ibadan, Ibadan, Nigeria; 2 University College Hospital, Ibadan, Nigeria; 3 Department of Epidemiology and Medical Statistics, Faculty of Public Health, College of Medicine, University of Ibadan, Ibadan, Nigeria

**Keywords:** Infant vaccination, Participatory learning, Urban slums, Experimental study

## Abstract

**Background:**

One of the strategies for improving vaccination uptake is to make communities understand the importance of immunization and this is expected to drive the demand for vaccines. Building the capacity of older women who supervise child care in Africa may improve infant vaccination in underserved communities. This study determined the impact of training of older women on their knowledge and support for infant vaccination in selected urban slum communities in Ibadan, Nigeria.

**Methods:**

This was a before-and-after study that enrolled women aged ≥35 years. They were trained with a manual and short video using participatory learning methods over an 8 month period. The content of their training includes importance of immunization timeliness and completion, how vaccines work and how to be advocates and supporters of infant vaccination. Their knowledge and support for infant vaccination at baseline were compared with post training values using Student’s t test and Chi square test with the level of significance set at 5%.

**Results:**

There were 109 women with mean age 55.8 ± 11.6. they had a mean of 5.7 ± 2.1 training sessions. At the end of the training, their knowledge about infant vaccination and the support they give to it increased from 4.8 ± 3.8 to 10.7 ± 0.6, and 3.1 ± 3.5 to 8.1 ± 1.7 respectively. Those with good knowledge about infant vaccination increased significantly from 37(33.9%) to 82(82.8%), while those with good support for the same increased from 31(28.4%) to 85(85.9%). Women who were ≤ 64 years significantly had improved knowledge after the training compared to the older ones. Those with post secondary education had better knowledge and greater support for infant vaccination at baseline. However, there was no difference in the knowledge and support for infant vaccination among the women across the different educational levels after the training.

**Conclusions:**

Participatory learning improved the knowledge about, and support for infant vaccination among older women supervising child care in these urban slum communities. Similar training may be extended to comparable settings in order to improve demand for infant vaccination.

## Background

Immunization is one of the cost-effective approaches being used in public health to improve child survival by preventing diseases that cause child morbidity and mortality [1]. It has transformed the trends of vaccine preventable diseases (VPD) globally with many of these diseases now under control, poliomyelitis about to be wiped out and smallpox already eradicated. Immunization has contributed immensely to the improvement of the quality of life in childhood as well as adulthood and its coverage is a good measure of the performance of the health system of a country [2]. It also has economic benefits as it prevents unnecessary spending on curative services, leading to conservation of resources [3] while increasing productivity through improvement in human cognitive and physical functions [4]. It is therefore, a priority for the health system, the family and other stakeholders to achieve and continue to maintain a high level of infant vaccination coverage in every country.

Infant vaccination coverage in Nigeria is still suboptimal and more infants in the families with the least wealth quintile are more likely to be unimmunized [5]. Meanwhile, concerted efforts have been made in the last decade to improve the vaccination coverage through improvement of vaccine infrastructures across the country and continuous training of health care and ad hoc staff in charge of vaccination [6, 7]. Traditional rulers, political leaders and community youths have also been involved in designing strategies to improve infant vaccination uptake [6]. A radical improvement in infant vaccination coverage Nigeria is imperative for Nigeria to achieve the third sustainable development goal. Hence, it is important to identify other channels through which infant vaccination coverage can be accelerated in order to realize this goal and ensure that the country reap the full benefit of good infant vaccination coverage as earlier stated.

In Africa and many developing countries, older women (comprising grandmothers, aunts, and even non-relatives in the family social circles) are expected to oversee the care of infants and mothers, a role that is well recognized and respected [8, 9]. This makes them great influencers in child care decisions. However, their influence is not recognized in childcare program and formal healthcare settings. Older women have been shown to influence decisions about infant feeding practices [10, 11], uptake of prevention of mother to child transmission of HIV services [12] and contraceptive use by mothers [13, 14]. They also influence the decision to seek health care services for infants [15] and this can include decision about uptake of vaccination. The influence these older women have can be harnessed to improve infant vaccination uptake in Nigeria and other developing countries but this can only become realistic if their capacity is developed to understand the importance of infant immunization. This can guide them when taking decisions about infant vaccination for the infants in their care, thus, fulfil the second strategic objective of the Global Vaccine Action Plan which is to make individual and communities to understand the importance of vaccines with the hope that this will drive the demand for vaccines [16]. It will also align with the mission of the Nigerian National Primary Care Development Agency (NPHCDA) [17] (the institution that coordinate vaccination activities in the country) to rapidly accelerate the vaccination coverage in Nigeria. In addition, it will contribute to the fulfilment of the vision of the NPHCDA to have a sustainable immunization service delivery through community driven strategies and the objective to sustain awareness about the importance of vaccination in the country [17]. This study therefore determined the impact of an intensive training of older women (35 years and above) on their knowledge and support for infant vaccination timeliness and completion among selected urban slum communities in Ibadan, southwest Nigeria.

## Methods

### Study area

This study was conducted in seven selected urban slum communities in Ibadan, a city located in Southwest Nigeria. These seven urban slums were located in Ibadan North and Ibadan North-East local government areas. Due to increased migration and prevailing poor economic condition in the country, there are many urban slum settlements in Ibadan and the study communities are parts of the inner-city slums which exist because of refusal of resettlement [18]. This is as a result of the strong attachment that the people have for the ancestral land in this part of the city [18]. Most of the slum dwellers are traders and the main religions practiced are Christianity and Islam. There were six health care centers in different locations within these communities that provided infant immunization services.

### Study population

The study population were women who were at least 35 years old and have the capacity to provide care for new born infants and their mothers in the selected communities.

### Study design

This was a before-and-after study design in which the baseline data on knowledge of and support for infant vaccination for each participant was taken and compared with the repeat data after the training, such that each participant acted as her own control.

### Study participants

The study participants were recruited over a 6 month period (July 2018 to January 2019) through the primary health care centres with the assistance of pregnant women who were living in selected urban slum communities in Ibadan, and were attending antenatal clinics at the time of the study. They were asked to bring along the potential care givers for their unborn babies to the clinic. In all, 109 older women were recruited consecutively over a six-month period.

### The training of older women in the communities

The older women were trained on a monthly basis over an eight-month period. A seven module training manual was used for the training sessions and the manual dwelt on the importance of infant vaccination and how older women can support the program. One module was taught every month and in the last month, there was a revision of all that had been taught from the manual. A short video was also used for video assisted teaching in the first four modules of the manual.

### The training materials

The manual and short video used for the training were developed based on findings from earlier interviews conducted among community members. The interviews included 22 Focused Group Discussions (FGDs) among women who were 35 years and above, six FGDs among community birth attendants and six in-depth interviews among traditional healers. The aim of the interview was to understand their views concerning infant vaccination timeliness and completion. The manual has seven modules and it uses different pedagogic methods to ensure participatory learning for adults. It covers different aspects of infant vaccination uptake including how vaccines work, the importance of timeliness and completion of infant vaccination, how to overcome barriers of infant vaccination, common myths about infant vaccination and how to be an advocate for infant vaccination. The training session for each module was designed to last for about 90 to 120 min. There were also assessment strategies for each module to determine how much learning had occurred.

The short video was used in the first four modules of the training manual and it was presented in Yoruba language (the prevalent local language in the study area). It emphasized the importance of infant vaccination, dispelled myths about infant vaccination and showed ways in which the barriers to infant vaccination can be handled.

### Data collection procedure

The data were collected using a semi-structured questionnaire designed with the aid of RedCap. There were English and Yoruba versions of the questionnaire but the Yoruba version was used for the data collection. The study instrument obtained data about sociodemographic characteristics, the knowledge about infant vaccination and support that the older women provide for infant vaccination. Eleven questions were used to assess knowledge and nine questions for support of infant vaccination. The data about the knowledge and support they provide for infant vaccination was repeated at the completion of the training modules.

### Data analysis

Data were captured with Red Cap and exported to SPSS v 25 for analysis. Each knowledge and support question were scored such that a correct response was scored 1 and an incorrect response scored 0. The overall mean scores were then calculated for the knowledge and support separately and the women’s scores were categorized as good knowledge or support when the score was greater than or equal to the 75th percentile, or poor knowledge or support when the score was below 75th percentile. Univariate analysis was done and frequencies and proportions were generated for relevant variable such as socio demographics of older women, level of knowledge and support for various vaccination activities. In addition, bivariate analysis was carried out using Students’ t test to determine the differences in the knowledge and support scores regarding infant vaccination pre and post intervention. Chi square test was used to identify factors associated with good/poor knowledge and good/poor support of vaccination activities at pre and post intervention. The statistical significance was set at 5%.

## Results

There were 109 older women who participated in the study. Their mean age was 55.8 ± 11.6 years and 32.1% of them were between 55 and 64 years age group. Many of them (56%) had five or more biological children and half (50.0%) had secondary school education. Table 1 shows the other details about their socio-demographics characteristics and their relationships with the pregnant women. The mean number of trainings that the older women had was 5.7 ± 2.1 trainings. Their mean knowledge about infant vaccination and mean support they give to infant vaccination in their communities at baseline were 4.8 ± 3.8 and 3.1 ± 3.5 respectively. Both improved significantly to 10.7 ± 0.6 and 8.1 ± 1.7 (*p* < 0.01 for both) at the end of the training period. Table 2 gives the details about the changes in the mean knowledge and support scores for the women from baseline to post-intervention.

**Table 1:** Sociodemographic characteristics of the older women

Selected sociodemographics	Frequency	Percentage
**Age group (years)**		
35–44	15	13.8
45–54	31	28.4
55–64	35	32.1
65 years and above	28	25.9
**Marital Status**		
Married	81	75.0
Widowed	26	23.9
Single	1	0.9
Separated	1	0.9
**Educational Level**		
No formal Education	29	26.6
Primary	22	20.4
Secondary	54	50.0
Tertiary	4	3.7
**Occupation**		
Business Woman/Trading	79	72.5
Artisan	10	9.2
Retired Civil Servant	5	4.6
Patent Medicine Vendor	1	0.9
Birth Attendant/Midwife	4	3.7
None	10	9.2
**Number of Children**		
1–2	8	7.3
3–4	40	36.7
5 and above	61	56.0
**Relationship of Older Woman to Mother**		
Mother-in-law	47	43.1
Mother	30	27.5
Sister	9	8.3
Sister-in-law	7	6.4
Aunt	6	5.5
Neighbor	6	5.5
Friend	2	1.8
Grandmother	2	1.8
**Total**	**109**	**100.0**

**Table 2:** Comparison of the older women’s mean scores for knowledge and support for infant vaccination

Scores	Mean ± SD	Mean Difference ± SD	95% CI	t-test	***p*** value
Knowledge score					
*Pre-intervention*	4.8 ± 3.8	5.9 ± 3.8	5.2–6.7	15.8	< 0.01
*Post-intervention*	10.7 ± 0.6				
Support score					
*Pre-intervention*	3.1 ± 3.5	4.9 ± 4.1	4.1–5.7	12.4	< 0.01
*Post-intervention*	8.1 ± 1.7				

At baseline, only 37(33.9%) had good knowledge about infant vaccination but this increased to 82(82.8%) post intervention. Also, only 31(28.4%) were supporting infant vaccination at baseline and this increased to 85(85.9%) after the training. Tables 3 and 4 show the association between selected demographic characteristics of the older women and the changes in their knowledge and support for infant vaccination respectively. There was no difference in the knowledge about infant vaccination among the different age groups at baseline, but after the intervention, those who were 64 years or younger significantly had improved knowledge compared with the older ones. Those who had secondary school education significantly had poor knowledge about infant vaccination at the baseline compared to others but post-training, there was no difference in the knowledge across the different level of educational attainment. However, these changes in knowledge were not affected by the number of children that the older women had nor their marital status. Regarding the support of the older women for infant vaccination at baseline, those with formal education significantly supported infant vaccination compared with those who did not have formal education. Also, the difference in their support for infant vaccination disappeared among the different educational attainment following the training. There was no association between the support of the older women for infant vaccination with respect to their age, number of children and marital status.

**Table 3:** Association between selected sociodemographic characteristics of the older women and their knowledge of infant vaccination

Selected sociodemographic characteristics	Good Knowledge n(%)	Poor Knowledge n(%)	Good Knowledge n(%)	Poor Knowledge n(%)
**Age group (years)**				
35–44	4 (26.7)	11 (73.3)	13 (92.9)	1 (7.1)
45–54	13 (41.9)	18 (58.1)	26 (89.7)	3 (10.3)
55–64	11 (31.4)	24 (68.6)	28 (87.5)	4 (12.5)
65 years and above	8 (28.6)	20 (71.4)	15 (62.5)	9 (37.5)
**Total**	**37 (33.9)**	**72 (66.1)**	**82 (82.8)**	**17 (17.2)**
	χ^2^ = 1.708	*p* = 0.78	χ^2^ = 9.50	*p* = 0.04
**Marital Status**				
Married	23 (28.4)	58 (71.6)	65 (84.4)	12 (15.6)
Single	0 (0.0)	1 (100.0)	1 (100.0)	0 (0.0)
Separated	1 (100.0)	0 (0.0)	1 (100.0)	0 (0.0)
Widowed	13 (50.0)	13 (50.0)	15 (75.0)	5 (25.0)
**Total**	**37 (33.9)**	**72 (66.1)**	**82 (82.8)**	**17 (17.2)**
	χ^2^ = 6.56	*p* = 0.08	χ^2^ = 1.41	*p* = 0.70
**Educational Level**				
No formal Education	12 (41.4)	17 (58.6)	22 (78.6)	6 (21.4)
Primary	10 (45.5)	12 (54.5)	15 (93.8)	1 (6.3)
Secondary	12 (22.2)	42 (77.8)	42 (82.4)	9 (17.6)
Tertiary	3 (75.1)	1 (25.0)	3 (75.0)	1 (25.0)
**Total**	**37 (33.9)**	**72 (66.1)**	**82 (82.8)**	**17 (17.2)**
	χ^2^ = 8.33	p = 0.04	χ^2^ = 0.58	*p* = 0.90
**Number of Children**				
1–2	2 (25.0)	6 (75.0)	7 (100.0)	0 (0.0)
3–4	12 (30.0)	28 (70.0)	27 (79.4)	7 (20.6)
5 and above	23 (37.7)	38 (62.3)	48 (82.8)	10 (17.2)
**Total**	**37 (33.9)**	**72 (66.1)**	**82 (82.8)**	**17 (17.2)**
	χ^2^ = 0.94	*p* = 0.62	χ^2^ = 1.73	*p* = 0.42

**Table 4:** Association between selected sociodemographic characteristics of the older women and their support of infant vaccination

	Pre intervention		Post intervention	
Selected sociodemographic characteristics	Good support n(%)	Poor support n(%)	Good support n(%)	Poor support n(%)
**Age group (years)**				
35–44	4 (26.7)	11 (73.3)	12 (85.7)	2 (14.3)
45–54	13 (41.9)	18 (58.1)	24 (82.8)	5 (17.2)
55–64	7 (20.0)	28 (80.0)	29 (90.6)	3 (9.4)
65 years and above	7 (25.0)	20 (75.0)	20 (83.3)	4 (16.7)
**Total**	**31 (28.4)**	**78 (71.6)**	**85 (85.9)**	**14 (14.1)**
	χ^2^ = 4.04	*p* = 0.40	χ^2^ = 2.09	*p* = 0.71
**Marital Status**				
Married	19 (23.5)	62 (76.5)	67 (87.0)	10 (13.0)
Single	1 (100.0)	0 (0.0)	1 (100.0)	0 (0.0)
Separated	0 (0.0)	1 (100.0)	1 (100.0)	0 (0.0)
Widowed	11 (42.3)	15 (57.5)	16 (80.0)	4 (20.0)
**Total**	**31 (28.4)**	**78 (71.6)**	**85 (85.9)**	**14 (14.1)**
	χ^2^ = 6.35	*p* = 0.09	χ^2^ = 0.97	*p* = 0.80
**Educational Level**				
No formal Education	9 (31.0)	20 (69.0)	24 (85.7)	4 (14.3)
Primary	11 (50.0)	11 (50.0)	15 (93.8)	1 (6.3)
Secondary	45 (83.3)	9 (16.7)	42 (82.4)	9 (17.6)
Tertiary	2 (50.0)	2 (50.0)	4 (100.0)	0 (0.0)
**Total**	**31 (28.4)**	**78 (71.6)**	**85 (85.9)**	**14 (14.1)**
	χ^2^ = 9.71	*p* = 0.02	χ^2^ = 1.99	*p* = 0.57
**Number of Children**				
1–2	2 (25.0)	6 (75.0)	6 (85.7)	1 (14.3)
3–4	8 (20.0)	32 (80.0)	27 (79.4)	7 (20.6)
5 and above	21 (34.4)	40 (65.6)	52 (89.7)	6 (10.3)
**Total**	**31 (28.4)**	**78 (71.6)**	**85 (85.9)**	**14 (14.1)**
	χ^2^ = 2.52	*p* = 0.28	χ^2^ = 1.85	*p* = 0.39

## Discussion

In this study, participatory learning was effective in improving the knowledge of infant vaccination and its support among older women who were involved in the care of infants in urban slums in Ibadan. This can greatly influence the way they handle the vaccination of infants they care for in their communities and likely improve the uptake of the same. Older women continue to be involved traditionally in decision making in child care and so it is important that they have correct information about infant vaccination which will help in their understanding of the importance of infant vaccines and help them able to ensure that the infants they care for have optimal vaccinations by demanding for it [16].

Participatory learning was the teaching method employed among these older women and this have been shown to promote effective learning and the learner is more likely to retain what is learnt [19]. This is because the learner is in charge of the learning process and the learning experience is based on the realities of the worldview of the learners. This makes the learning process familiar to the older women as things they can readily relate with and their experiences were employed in the teaching process. The older women also worked in teams which would have fostered socialization among them [20], encouraged their learning and boosted their confidence at building skills on how to support infant vaccination. Edutainment was also used for the first four sessions of training and this teaching method is useful among less literate population as seen in this study group where almost half of the participants either had only primary education or did not have any formal education. This made the teaching process more interesting and comprehension easier for them.

Clearly, higher educational attainment was significantly associated with better knowledge about infant vaccination and its support at baseline and this have been a common finding in earlier researches. However, this association was no longer significant at the end of the training and this means that the training bridged the gap of knowledge between the well-educated older women and those who did not have much education. Thus, the training of these older women about infant immunization produced faster results in a short term compared with enrolling them in formal education. While having a formal education would have been more desirable as it will have a more far reaching effect in the lives of these women and their communities, it was less feasible in their current situation due to various factors like life expectations, competing responsibilities and cost. The type of training used in this study is an inexpensive way to improve infant vaccination and uptake of other health interventions in a short term which could improve the quality of life in slum communities.

Although the knowledge about infant vaccination improved significantly after the training, the women who were 65 years and above significantly did not have much improvement in their knowledge like the other age group. It is possible that they had some morbidities which are associated with aging that could impair learning [21]. These morbidities could include hearing and visual deficits which can make the learning process difficult [21]. There was however no screening for such defects before the participants were recruited. Aside these, older adults have been shown to process new information slower than the younger adults as a result of aging [22]. This is likely to be worse when there is multiple new information to be processed as seen in this study. They might require more teaching hours compared to others or a completely different curriculum structure for them to achieve the same improvement in their knowledge about infant vaccination as seen in the other age groups [23].

The skills gained from the training by these older women would likely have a ripple effect in the demand for infant immunization in their communities in the long run as the training built their capacity to be advocates for infant vaccination. This is in line with the recommended action to increase understanding and demand for vaccines. The training will likely impact on the vaccination of the infants they will provide care for, including the quality of their contribution in the decision-making process for uptake of infant vaccination both at the household and community level. They are also likely to have impact on infant immunization in their communities because of the influence they weld and the respect they command.

The definition of an older woman in this study was a woman who was 35 years or older because of the instability associated with urban slum settings where poor literacy and poverty are common and these make girls start to have children at younger ages than what is seen in the other parts of the communities. This is evident in the fact that majority of the participants were either mothers or mother-in-law to the pregnant women whose infants they were to care for after delivery. This underscores the importance of adjustment of the cut off age if this model of training of older women is to be replicated in other settings. This strategy of training older women about infant immunization with the aim of improving infant vaccination can be replicated in settings where older women are involved in decision making about infant care. The skills they will gain from such training will guide them in taking good decisions about the vaccination of the infants they provide care for and it can result in optimal infant vaccination. The set up where older women supervise the care of infants is present in many developing countries as earlier mentioned.

There are some limitations in this study which should be mentioned. The before-and-after study design that was used in this study is characterized by threats to internal validity so steps were taken to control these threats. The same questionnaire was used to collect data both at the baseline and at the end of the training by research assistants who were fluent in Yoruba language. The data in this study was obtained from the self-report of the older women and this is prone to social desirability bias which can make them over report their knowledge and support for infant vaccination at baseline and post-training. Asking them the same question after the training might also influence their responses which may not reflect their true support for infant vaccination. Also, there might be external factors which affected their knowledge and support for infant vaccination apart from the training they had but we were not aware of any concurrent program which could have had such effect. Lastly, none of the women dropped out of the study all through the training period. This study was also conducted only in selected communities in southwest Nigeria which may limit the application of this strategy outside these settings. However, the culture of older women supervising infant care is widespread in Nigeria, many African communities and some developing countries. Therefore, this strategy may improve the knowledge and support that older women have for infant vaccination.

## Conclusions

In conclusion, this study showed that the capacity of older women involved in care of infants in urban slums in Nigeria can be built for increased understanding of infant immunization and support through participatory learning even though this was less apparent among the elderly among them. This can be a useful strategy in improving infant vaccination uptake in such disadvantaged settings.

## Data Availability

The datasets used and/or analyzed during the current study are available from the corresponding author on reasonable request.

## Abbreviations

FGDs: Focused Group Discussions
HIV: Human Immunodeficiency Virus
NPHCDA: National Primary Care Development Agency
PHC: Primary Health Center
SPSS: Statistical Package for Social Sciences
VPD: Vaccine Preventable Diseases
